# An Unusual Case of Bleeding: Acquired Hemophilia A

**DOI:** 10.7759/cureus.45577

**Published:** 2023-09-19

**Authors:** Omeed S Jahangiri, Michael P Wurzer, Mohammad Malik

**Affiliations:** 1 Department of Clinical Medicine/Internal Medicine, Burrell College of Osteopathic Medicine, Las Cruces, USA

**Keywords:** internal medicine, critically ill, hematology, coagulation, bleeding disorder, clinical case report, factor viii and factor viii inhibitors, hemato-oncology, acquired hemophilia a (aha)

## Abstract

Acquired hemophilia A (AHA) is a bleeding disorder, autoimmune in nature, in which the body produces IgG antibody inhibitors that attack coagulation factor VIII, causing deficiency. It is largely seen in the elderly, but most cases are idiopathic. Cases of acquired hemophilia A can occur in the presence of neutrophilia, infection, acute physiological stress, medication effect, tissue necrosis, various inflammatory disorders, and/or malignancy, which presents a formidable challenge with clinical workup. This case illustrates the potential for a masked bleeding disorder in a complex elderly male patient and the value of a thorough history-taking and workup. Although rare, acquired hemophilia recognition is essential for appropriate therapies to be started as early as possible and for cases to not easily be confused for another bleeding disorder in an acute care setting after ruling out other acute/common causes of similarly presenting symptoms.

## Introduction

Acquired hemophilia A (AHA) is an autoimmune disorder through which the body begins to produce autoantibodies to coagulation factor VIII resulting in a factor VIII deficiency. It is considered a rare disorder with an incidence of one and a half cases per million persons per year [1]. This rarity along with the acquired nature of the disorder makes diagnosis quite difficult. AHA is more prevalent in persons greater than 65 years old but also appears in younger populations in association with the postpartum period, malignancy, and a history of other autoimmune disorders such as rheumatoid arthritis and systemic lupus erythematosus (SLE). The mechanisms by which the disease is acquired are not well understood, with approximately 50% of cases being considered idiopathic [2]. The purpose of this case study is to highlight the importance of considering such coagulation disorders in the presentation of new-onset bleeding without clear risk factors. Following the diagnosis of such disorders, further testing should be conducted in an attempt to find the underlying cause, both for the benefit of the patient and for the understanding of the disease’s pathogenesis.

## Case presentation

A 68-year-old male presented as a transfer from an outside hospital for a chief concern of anemia with secondary bleeding, bruising, and syncopal episodes with falls. These syncopal episodes were not preceded by chest pain or dizziness. His past medical history was significant for coronary artery disease with stent placement in 2006, alcohol abuse, and chronic opioid dependence. He was previously on aspirin therapy. However, it was discontinued approximately one week prior to his arrival during a visit to his primary care provider. At that time, he endorsed new-onset bruising and falls, which had occurred over the past few weeks leading up to his visit. He stopped taking the medication two days prior to arrival.

**Figure 1:**
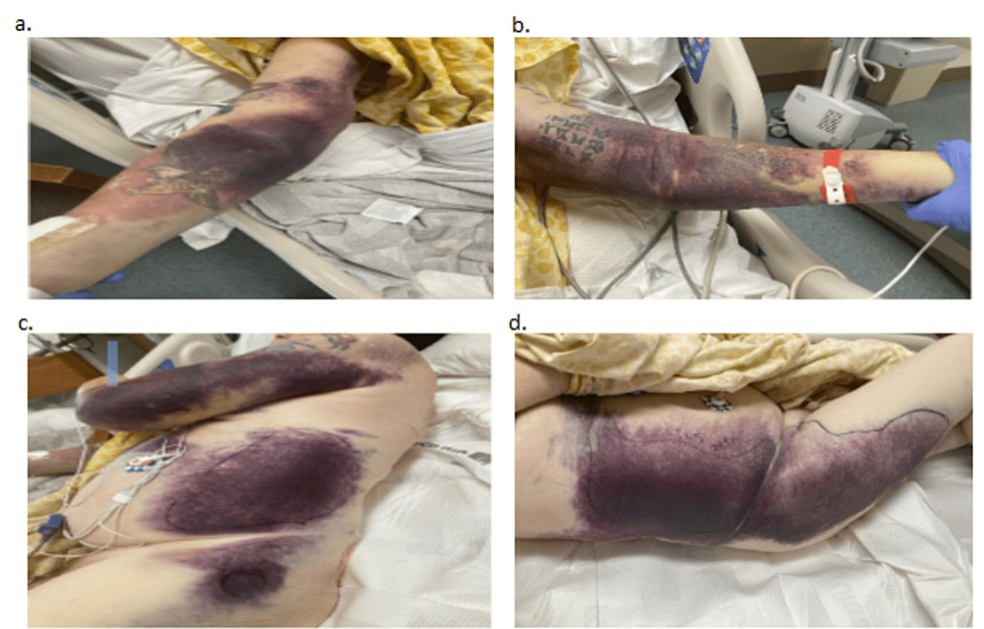
Images of the Patient’s Sites of Ecchymosis a: right arm, b: left arm, c: left side/torso, d: right side/torso

On initial evaluation, the patient had active bleeding from a right internal jugular central venous access site, abdominal pain, hip pain, and extensive ecchymosis throughout his body. The patient was unsure how he had obtained the ecchymotic regions on his body and ultimately attributed them to his falls. The extent of ecchymosis is depicted in Figure 1.

**Figure 2:**
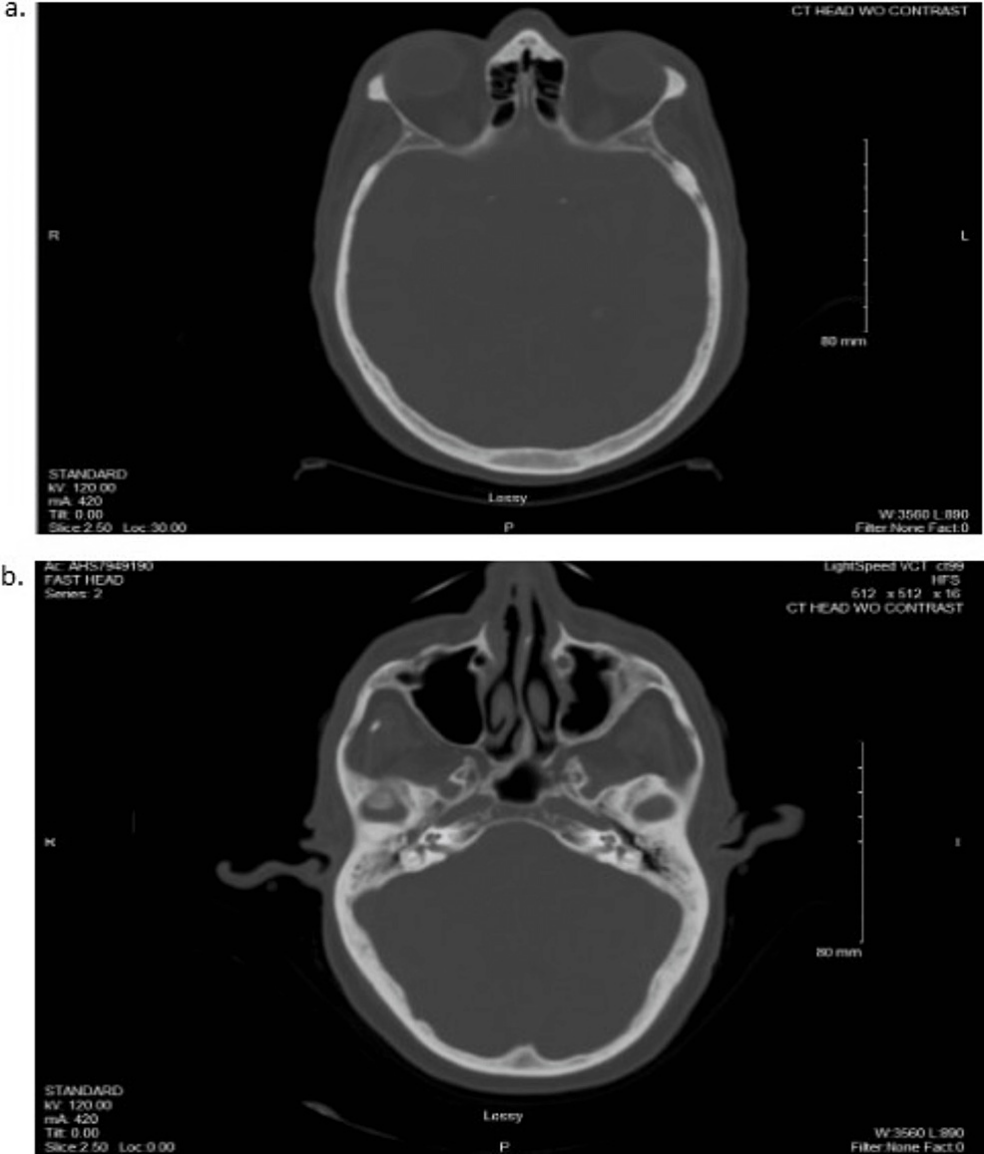
CT of the Head Without Contrast a: transverse plane, caudal; b: transverse plane, rostral CT: computed tomography

**Figure 3:**
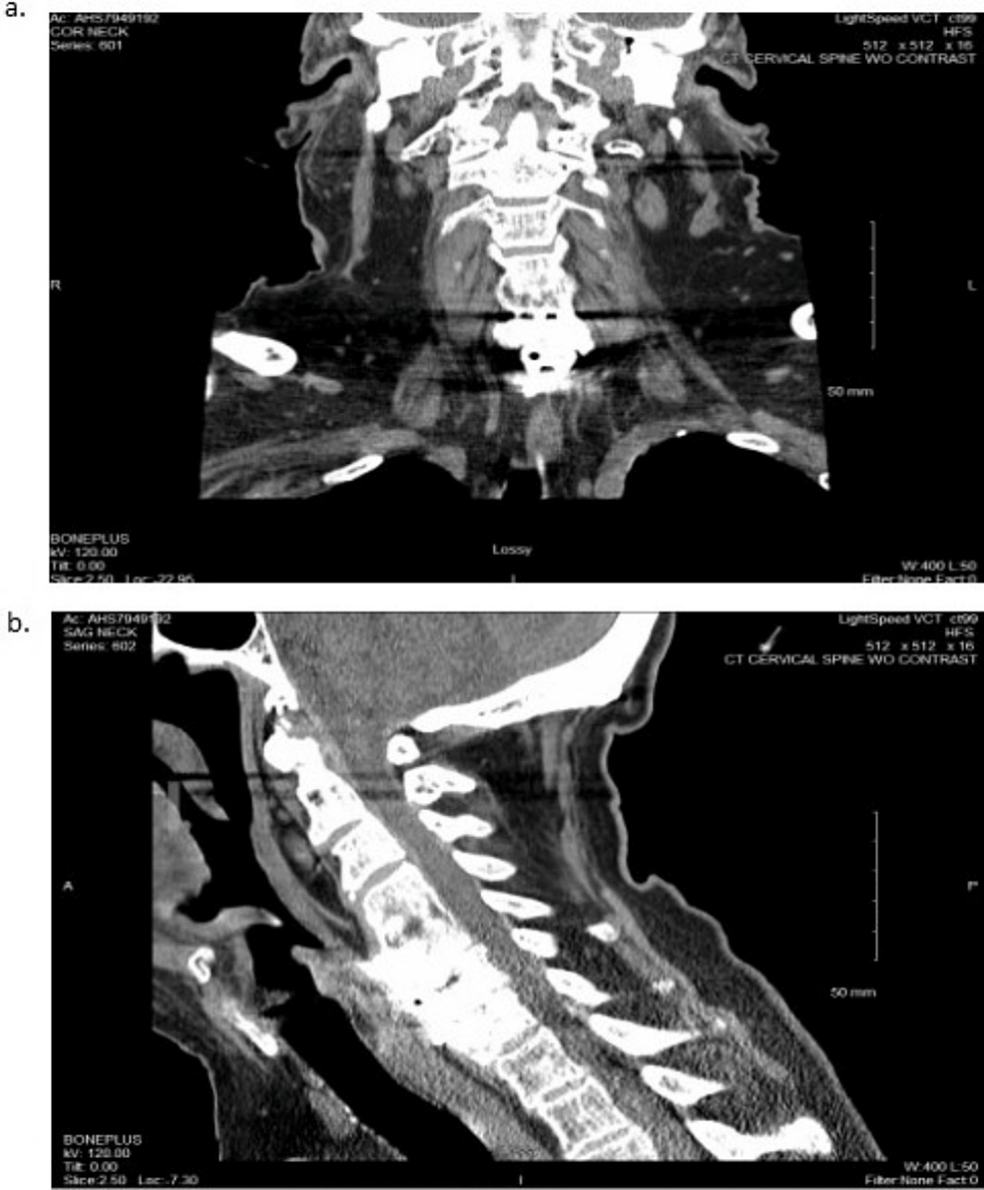
CT of the Cervical Spine Without Contrast a: coronal plane, b: sagittal plane CT: computed tomography

**Figure 4:**
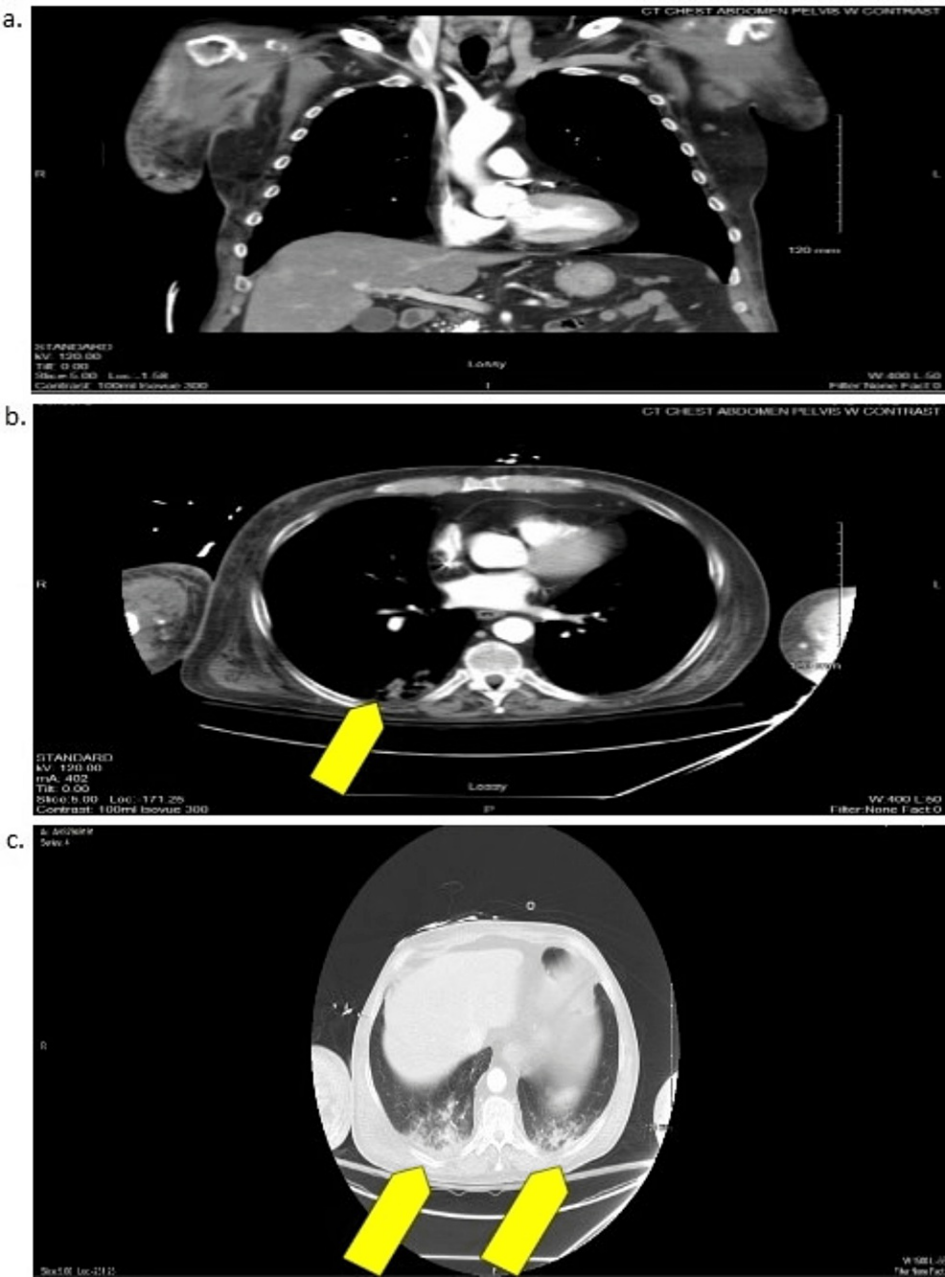
CT of the Chest With Contrast a: mid-coronal plane; b: mid, upper-transverse plane (yellow arrow indicates the area of consolidation); c: lower transverse plane, chest CT lung view (yellow arrows indicate areas of consolidation) CT: computed tomography

**Figure 5:**
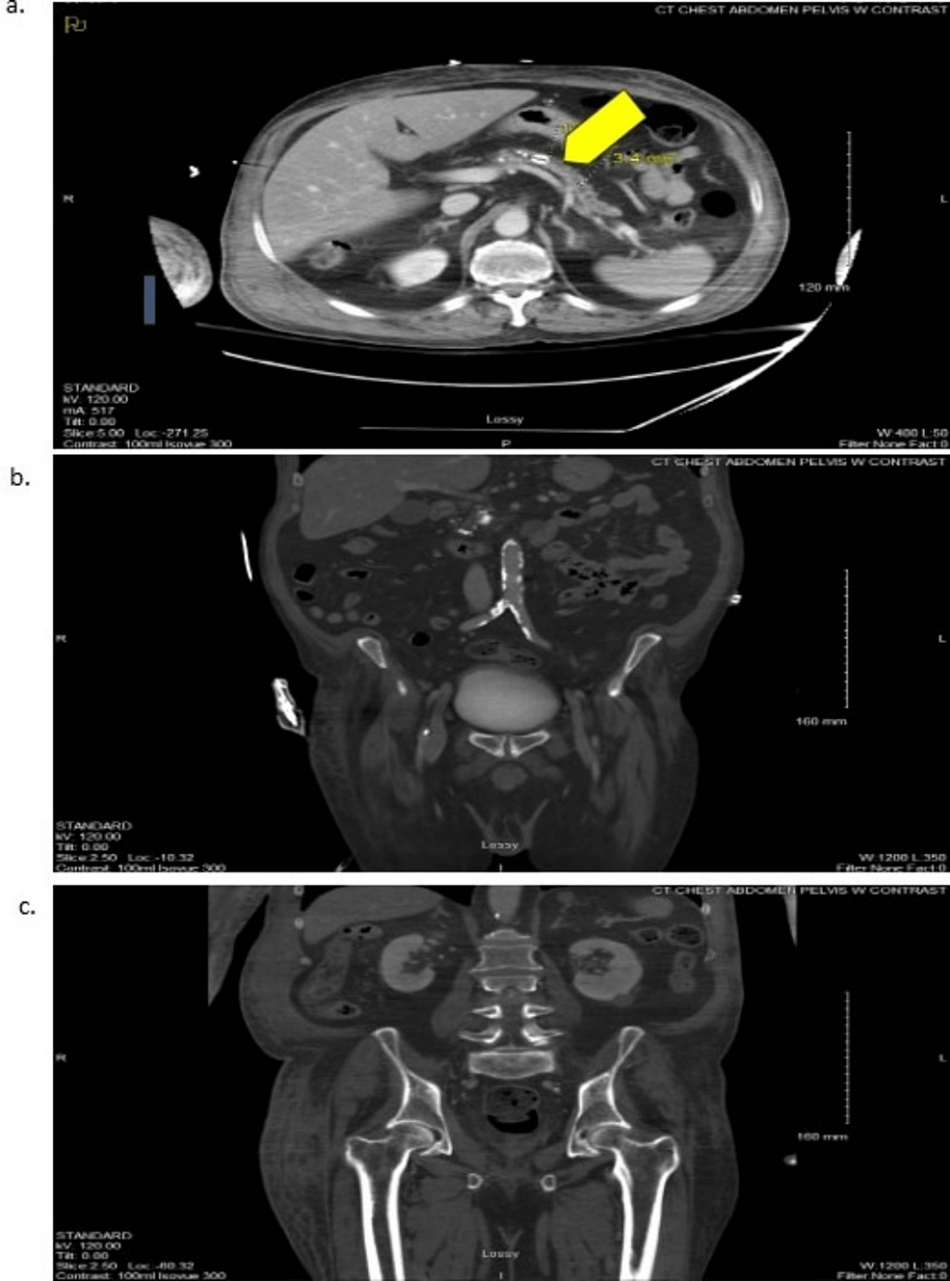
CT of the Abdomen and Pelvis With Contrast a: transverse plane, caudal (yellow arrow indicates pancreatic duct stone); b: coronal plane, caudal; c: coronal plane, rostral CT: computed tomography

He had no prior history of easy bleeding or bruising, and he denied fever, chills, and abnormal weight loss. Computed tomography (CT) without contrast of the head and CT with contrast of the cervical spine, chest, abdomen, and pelvis were ordered to explore for internal hemorrhage, such as hemarthrosis, muscle bleeding, or organ bleeding (Figures 2-5).

**Figure 6:**
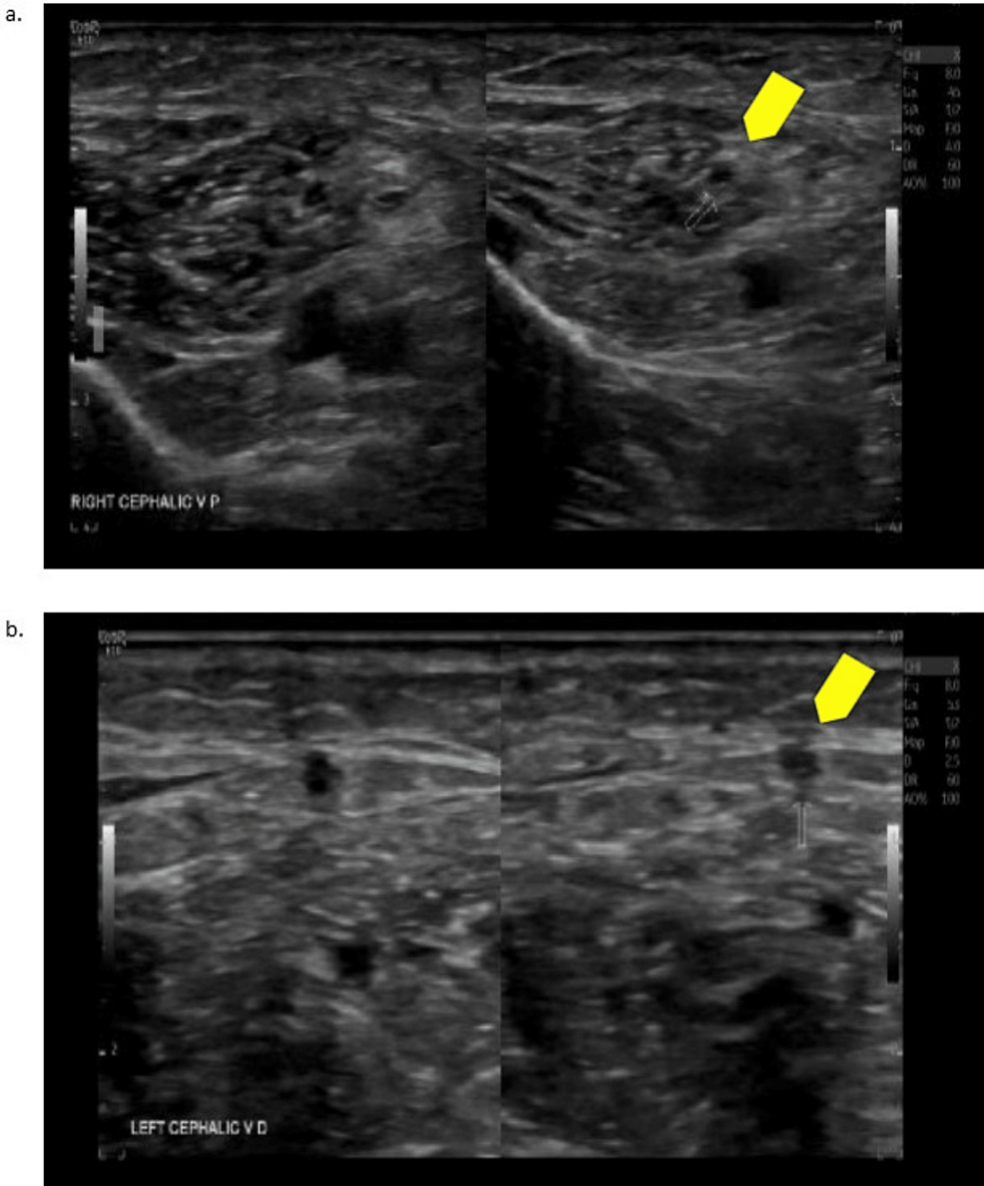
Ultrasounds of Bilateral Cephalic Veins a: right cephalic vein (yellow arrow indicates thrombus), b: left cephalic vein (yellow arrow indicates thrombus)

No bleeding was found, but imaging did show evidence of pneumonia as small consolidations in bilateral lung bases and a pancreatic duct stone. An upper extremity ultrasound was also routinely obtained due to the patient’s longer stay and immobility at our hospital, which showed bilateral cephalic vein thrombosis (Figure 6).

The patient previously had a hemoglobin of 5 g/dL at the outside hospital where he received two units of packed red blood cells (PRBCs). Table 1 provides pertinent and abnormal laboratory values.

**Table 1:** Pertinent Laboratory Values Abnormal laboratory values are marked as ***. Normal reference ranges are alongside each abnormal laboratory value descriptor. MCV: mean corpuscular volume, TIBC: total iron-binding capacity, ALT: alanine transaminase, AST: aspartate aminotransferase, LDH: lactate dehydrogenase, aPTT: activated partial thromboplastin time, PT: prothrombin time, INR: international normalized ratio

Hemoglobin (13.5-17.7 g/dL)	10.4→7.8 g/dL ***	Ristocetin cofactor	>350%
MCV	92.3 fL	Anticoagulation factor VIII (0 BU)	113.5 BU ***
Platelet count	150 k/uL	Factor VIII activity (43.2%-159.3%)	1.5% ***
Reticulocytes (0.30-2.40 ug/dL)	3.52% ***	Fibrinogen	323 mg/dL
Total iron (61-157 ug/dL)	27 ug/dL ***	Total bilirubin (0.2-1.0 mg/dL)	1.4 umol/L ***
TIBC (250-450 ug/dL)	116 ug/dL ***	Alkaline phosphatase	37 U/L
Ferritin	379 ng/mL	ALT	9 U/L
Haptoglobin (30-120 mg/dL)	190 mg/dL ***	AST	11 U/L
LDH	184 U/L	aPTT (26-38 seconds)	92 seconds ***
Vitamin B12	393 pg/mL	PT	12.8 seconds
Folate	4.8 ng/mL	INR	1.1
D-dimer (less than 500 ng/mL)	538 ng/mL ***	von Willebrand antigen (46%-178%)	227.3% ***

His hemoglobin on arrival at our hospital was 10.4 g/dL, although the hemoglobin decreased to 7.8 g/dL within 24 hours following his presentation. Initial complete blood count (CBC) test also showed a mean corpuscular volume (MCV) of 92.3 fL, platelets of 150 k/uL, and reticulocytes of 3.52%. Iron studies demonstrated a total iron of 27 ug/dL, total iron-binding capacity (TIBC) of 116 ug/dL, and ferritin level of 379 ng/mL. Further blood work was obtained to evaluate for hemolysis, including a haptoglobin of 190 mg/dL and lactate dehydrogenase (LDH) of 184 U/L. Vitamin B12 and folate levels were within normal limits. The liver function panel was significant for a total bilirubin of 1.4 umol/L with all other values being within normal limits. This mild bilirubin elevation was thought to be the result of mild transfusion-related hemolysis. Although one would expect haptoglobin to be decreased and LDH to be increased in intravascular hemolytic reactions, it is known that immature platelet fraction (IPF) was within normal range, and blood smear showed normochromic normocytic anemia with some polychromasia, neutrophilia, and slightly decreased platelets. A disseminated intravascular coagulation (DIC) profile was also obtained and was significant for an elevated aPTT of 92 seconds, elevated D-dimer of 538 ng/mL, and normal PT, INR, and fibrinogen. A mixing study was then ordered to further investigate the etiology of this elevated aPTT, and this demonstrated no correction. Due to the result of the mixing study testing for von Willebrand antigen, ristocetin cofactor, anticoagulation factor VIII, and factor VIII levels were ordered. The resulting laboratory work was significant for a normal ristocetin cofactor level, elevated von Willebrand antigen of 227.3%, an elevated factor VIII inhibitor level of 113.5 BU, and decreased coagulation factor VIII activity of 1.5%. The patient was ultimately given 3,500 units of factor VIII inhibitor bypass activity (FEIBA) and transferred to the nearest hemophilia center with a presumptive diagnosis of acquired hemophilia A.

To note, haptoglobin production and release is increased in the presence of growth hormone, insulin, bacterial endotoxins, and pro-inflammatory cytokines such as interleukin factor 1 (IL-1), interleukin factor 6 (IL-6), and tumor necrosis factor-α (TNF-α) [3]. The patient presented in a very ill state, most likely more malnourished than the average patient, and his additional evidence of pancreatic duct stone and pneumonia could have “elevated” his haptoglobin to seem normal or elevated, although decreased with his hemolysis. Additionally, one would expect the haptoglobin level to be increased in serum during intravascular hemolysis. However, autoimmune hemolytic anemia tends to cause extravascular hemolysis. With extravascular hemolysis, it is less likely to observe a rise in haptoglobin levels because there would be no hemolysis or products of hemolysis intravascularly versus hemolysis occurring in locations such as the spleen [4,5]. In the big picture of the patient, his autoimmune hemolytic anemia most likely masqueraded the small effects of transfusion-related hemolysis.

## Discussion

AHA most often presents with subcutaneous bleeding, which occurs in over 80% of cases. Other presentations include muscular bleeding (>40%) and gastrointestinal bleeding (>20%), followed by genitourinary and retroperitoneal bleeding in less than 10% of cases. It is worth noting that hemarthrosis, a common presentation in congenital hemophilia A, is a rare presentation in the acquired form [6]. AHA is diagnosed via coagulation and mixing studies as shown in the patient’s case. Initial laboratory findings demonstrate a prolonged bleeding time with an elevated aPTT and normal PT as coagulation factor VIII is part of the intrinsic pathway. With this, a mixing study is then performed where the patient’s blood is mixed with normal plasma at a 1:1 ratio, and the aPTT is measured immediately after the mix, as well as after one hour of incubation [7]. If this mixing corrects the values of the initial coagulation tests, this is indicative of a simple coagulation factor deficiency as the normal plasma is able to supply an appropriate amount of coagulation factor on its own to overcome the patient’s deficiency. If the mixing study does not correct the initial tests, this is indicative of an inhibitor being present, such is the case in AHA. The normal plasma is unable to correct the aPTT as the inhibitor in the patient’s blood is able to continually impede coagulation despite the presence of an appropriate amount of coagulation factors. These coagulation factor inhibitors essentially attach to the coagulation factors and prevent them from activating/working, thus causing them to have zero effect compared to normally flowing freely without any “blockade” on their function. The final step is to distinguish what type of inhibitor is present by testing for levels of specific inhibitors. Examples of other inhibitors include lupus anticoagulant and anticoagulation factor VII.

The treatment of AHA is centered around first achieving hemostasis by either replacing coagulation factor VIII or using bypassing agents, followed by eradicating the inhibitor. Replacement of coagulation factor VIII with human factor or desmopressin has been shown to only be efficacious when low levels of inhibitor are present. Inhibitor levels are measured using Bethesda units. One Bethesda unit is defined as the amount of inhibitor in a plasma sample that will neutralize 50% of one unit of coagulation factor VIII in normal plasma after a two-hour incubation period at 37°C. It is important to note that coagulation factor VIII inhibitors have been shown to exhibit both first- and second-order kinetics. Therefore, a linear relationship is not always seen when calculating the level of factor VIII inhibition [8]. At five or greater Bethesda units, it is recommended to instead treat with a bypassing agent such as activated prothrombin complex concentrate or recombinant activated coagulation factor VII. Bypassing agents work by bypassing the need for coagulation factor VIII in the production of thrombin. After hemostasis is achieved, inhibitor eradication should be initiated via the use of corticosteroids and immunosuppressants such as cyclophosphamide or rituximab. Studies have demonstrated that treatment with prednisone 1 mg/kg/day and cyclophosphamide 50-100 mg/day is the most effective, with rituximab being reserved as a second-line agent for cases where the patient is intolerant or resistant to cyclophosphamide. The goal of treatment is to achieve an undetectable amount of autoantibodies (less than 0.6 BU) and coagulation factor VIII levels of greater than 50% [9].

Amu-Hernández et al. produced two case reports from May 2023, showing two different presentations of acquired hemophilia A [10]. In the first case, a 72-year-old male with no recent traumatic history or prior history of bleeding presented with a two-month history of large hematomas. His condition worsened when he suddenly stopped taking methylprednisolone that he was taking for omalgia without tapering. He was successfully treated with cyclophosphamide, rituximab, and intravenous immunoglobulin (IVIG) for five days due to nonresponse to treatment with methylprednisolone. Tranexamic acid and recombinant factor VII (later replaced with activated prothrombin complex concentrate) were also used. Full treatment was seven weeks with 19 packed red blood cells transfused overall. The second case was a 73-year-old female with a history of vitiligo, who had been diagnosed with pulmonary embolism and placed on warfarin anticoagulation. She subsequently developed uncontrolled epistaxis that persisted even when her anticoagulation was withdrawn. In her case, successful treatment was achieved with corticosteroid, bypassing agent, and tranexamic acid without the need for any blood transfusion. These cases highlight the similarities and differences between patient presentation and objective findings, further establishing why “cause and effect” is not always so clear in these cases. Our patient presented in a more critical condition with many more secondary findings, making the case a lot less straightforward than most. Ideally, the efficiency at finding a cause could be better, but the challenge of presentation, setting, and costs of diagnostics can diminish efficiency. In a critically ill patient with no other explainable causes of bleeding, providers should identify the workup needed to rule out acquired hemophilia.

## Conclusions

Most clinicians are able to recognize common causes of bleeding disorders. However, they should also be familiar with more obscure causes such as acquired hemophilia A. When a patient’s history and clinical workup raise questions concerning the etiology of their bleeding, more rare disorders should be considered. Timely diagnosis and management of these conditions is crucial and serves to improve the prognosis for the patient. Ultimately, once the patient’s condition has stabilized, further workup should be initiated to investigate the underlying source of their newly acquired bleeding disorder.

## References

[1] Franchini M, Vaglio S, Marano G, Mengoli C, Gentili S, Pupella S, Liumbruno GM (2017). Acquired hemophilia A: a review of recent data and new therapeutic options. Hematology.

[2] Collins PW, Hirsch S, Baglin TP (2007). Acquired hemophilia A in the United Kingdom: a 2-year national surveillance study by the United Kingdom Haemophilia Centre Doctors' Organisation. Blood.

[3] MacKellar M, Vigerust DJ (2016). Role of haptoglobin in health and disease: a focus on diabetes. Clin Diabetes.

[4] Yang F, Ghio AJ, Herbert DC, Weaker FJ, Walter CA, Coalson JJ (2000). Pulmonary expression of the human haptoglobin gene. Am J Respir Cell Mol Biol.

[5] Wang Y, Kinzie E, Berger FG, Lim SK, Baumann H (2001). Haptoglobin, an inflammation-inducible plasma protein. Redox Rep.

[6] Lombardi M, Cardenas AC (2023). Hemarthrosis. StatPearls Publishing.

[7] Paustian Paustian, T. T., Heesun-Rogers Heesun-Rogers, J. J., & Kottke-Marchant, K. (2012, January 16 (2023). Technical brief: Mixing study, incubated APTT. https://portals.clevelandclinic.org/portals/66/PDF/techbriefs/TB_MixingStudyIncubatedAPTT.pdf.

[8] Ma AD, Carrizosa D (2006). Acquired factor VIII inhibitors: pathophysiology and treatment. Hematology Am Soc Hematol Educ Program.

[9] Janbain M, Leissinger CA, Kruse-Jarres R (2015). Acquired hemophilia A: emerging treatment options. J Blood Med.

[10] Amu-Hernández LA, Marzo-Alonso C, Tugues-Peiró A, Vicente-Pascual EP, Monteagudo-Aguilar P (2023). A case report of idiopathic acquired hemophilia type A. Cureus.

